# The Role of Erythropoietin in Preventing Anemia in the Premature Neonate

**DOI:** 10.3390/children10121843

**Published:** 2023-11-24

**Authors:** Oana Cristina Costescu, Eugen Radu Boia, Marioara Boia, Daniela Mariana Cioboata, Florina Marinela Doandes, Nicoleta Lungu, Mihai Dinu, Emil Radu Iacob, Aniko Maria Manea

**Affiliations:** 1 Department of Neonatology, “Victor Babes” University of Medicine and Pharmacy Timisoara, Eftimie Murgu Square 2, 300041 Timisoara, Romania; costescu.oana@umft.ro (O.C.C.); boia.marioara@umft.ro (M.B.); cioboata.daniela@umft.ro (D.M.C.); doandes.florina@umft.ro (F.M.D.); lungu.nicoleta@umft.ro (N.L.); radueiacob@umft.ro (E.R.I.); manea.aniko@umft.ro (A.M.M.); 2 ENT Department, “Victor Babes” University of Medicine and Pharmacy Timisoara, Eftimie Murgu Square 2, No. 2, 300041 Timisoara, Romania; 3 PhD School Department, “Victor Babes” University of Medicine and Pharmacy Timisoara, Eftimie Murgu Square 2, 300041 Timisoara, Romania; 4 Faculty of Medical Engineering, University “Politehnica” of Bucharest, Gheorghe Polizu Street, No. 1–7, 011061 Bucharest, Romania; mihai.dinu1411@stud.fim.upb.ro; 5 Department of Pediatric Surgery, “Victor Babes” University of Medicine and Pharmacy, Eftimie Murgu Square 2, 300041 Timisoara, Romania

**Keywords:** erythropoietin, EPO, rhEPO, anemia, anemia of prematurity, AOP, erythrocyte transfusions, RBC transfusions

## Abstract

Recombinant human erythropoietin (rhEPO) treatment is an alternative to red blood cell (RBC) transfusions in neonates presenting anemia of prematurity (AOP). This study assesses the impact of early rhEPO administration on AOP (any stage) incidence, as well as the incidence of individual AOP stages and RBC transfusions. Out of 108 preterm neonates, 49 were administered rhEPO and compared to the remaining group using univariate and multivariate analyses. Univariately, gestational age (GA), birth weight (BW), hemoglobin (Hb), hematocrit (HCT), RBC levels, and iron administration were significantly associated with AOP (*p* < 0.05 each); however, only the latter remained significant following multivariate analysis (AOR: 2.75, 95% CI, 1.06–7.11). Multinomial analysis revealed rhEPO treatment was associated with a near three-fold reduction in moderate AOP incidence (OR: 0.36, 95% CI, 0.15–0.89). Furthermore, ANCOVA revealed positive correlations between rhEPO administration and 21-day Hb (*p* < 0.01), HCT (*p* < 0.05), and EPO (*p* < 0.001) levels. The results confirm previously reported benefits of rhEPO treatment, such as reduced moderate AOP incidence and increased Hb, HCT, and serum EPO levels.

## 1. Introduction

Anemia of prematurity (AOP) is a hypo-regenerative, normocytic, normochromic anemia that appears between the second and sixth weeks of life and is characterized by multiple factors, including reduced serum erythropoietin (EPO) levels, iatrogenic blood loss, hemorrhage, hemolysis, hypovolemia, insufficient erythropoiesis, and reduced red blood cell (RBC) survival [1,2].

AOP primarily affects preterm neonates with gestational ages (GA) under 35 weeks and can be caused by various other physiologic and non-physiologic factors. The liver is the main site of EPO production; it is less sensitive to anemia and hypoxia and slower in responding to hematocrit (HCT) level decreases in comparison to the kidney and phlebotomy blood loss following laboratory tests, which are more frequently performed in preterm than in full-term neonates [3,4,5]. Its high incidence rate, symptomatology, and association with increased need for transfusions make it a problematic disease in pediatric practice [3]. Furthermore, RBC transfusion administration thresholds are reportedly lower in clinal practice compared to the guidelines, which, alongside an increase in morbidity and mortality, prompts further research into the optimization of perioperative strategies [6].

In combination with hypoxia and hypotension, AOP forms a triad that has been correlated with high mortality at 30 days of life and, unfortunately, predominantly affects premature newborns, which are at high risk of morbidity and mortality and prone to perioperative critical events [7,8].

AOP has traditionally been treated using RBC transfusion; however, in the past 40 years, EPO has become both an important pathophysiological characteristic of the disease and a treatment for it, in the form of recombinant human EPO (rhEPO) [1,9,10,11,12,13,14].

Data have shown that rhEPO stimulates erythropoiesis and decreases both the dosage and frequency of RBC transfusions [1]. Most reports indicate higher per-kilogram doses are more effective in preterm neonates, as they present increased plasma clearance and distribution volume compared to full-term newborns [2].

Although there is extensive research on the efficacy and side effects of rEPO administration, there is not yet a consensus in regard to whether 7-day or 21-day administration is more effective when treating AOP, with some evidence showing that both contribute to increased reticulocyte counts and hemoglobin (Hb) levels; however, only the latter has been shown to simultaneously reduce the number of neonates requiring transfusion [9]. Furthermore, a Cochrane systematic review [15] revealed that early administration of rhEPO and other erythropoiesis-stimulating agents has been associated with a reduction in the number of required transfusions, neurological impairments between the 18th and 22nd months of life, intraventricular hemorrhage (IVH), and periventricular leukomalacia (PVL), as well as an increase in Bayley-II Mental Development Index (MDI) scores between the 18th and 22nd months of life. Multiple systematic reviews have also reported a reduction in the incidence of necrotizing enterocolitis (any stage) following rhEPO treatment, while finding no significant relationship to mortality or bronchopulmonary dysplasia [15,16,17]. Further, there has been no significant association between early or late rhEPO administration and retinopathy of prematurity (ROP) following four recent systematic reviews [15,16,18,19]. 

The main aim of this non-randomized controlled trial is to assess the effects of rhEPO administration in the first week of life on AOP (any stage) incidence rate at 21 days of life, different levels of AOP severity (i.e., mild, moderate, and severe), and RBC transfusions. Moreover, this experimental study examines the impact of early rhEPO administration on Hb, HCT, and serum EPO levels at 3 weeks of life. 

## 2. Materials and Methods

### 2.1. Study Population and Methodology

This study includes 108 neonates admitted to the Neonatology and Preterm Department of the “Louis Turcanu” Children’s Emergency Clinical Hospital in Timisoara, Romania during October 2021 and December 2022. The inclusion criteria included GA under 34 weeks and BW under 2500 g. The exclusion criteria included congenital cardiac, neurological and renal malformations; genetic syndromes; congenital infections; and individual or family hematological diseases, such as hemolytic anemia due to group and Rh isoimmunization, microspherocytosis, and thalassemia. The GA was estimated using the last menstrual period. Birth weight (BW), alongside 1 and 5 min Apgar scores, were measured during the first postnatal minutes. Prothrombin Time (PT) and activated Partial Thromboplastin Time (aPTT) were measured during the first day of life in order to analyze a potential correlation to AOP or one of its stages. Lactate dehydrogenase (LDH) levels were measured within the first six postnatal hours. Hb, HCT, RBC, and serum EPO levels were measured at 1, 7, and 21 days of life. Given that the administration of prophylactic rhEPO is very important for neonates with low serum EPO levels, randomization could not be performed, and the intervention was performed in neonates who presented EPO levels under 4.3 mUI/mL on the first of life. Therefore, the intervention group consisted of 49 neonates that were administered rhEPO-beta prophylactically subcutaneously at a dose of 500 U/kg BW at 2, 4, 7, 14, 21, and 28 days of life and, if required, they also received RBC transfusions. The remaining 59 neonates that were not administered rhEPO formed the control group. According to the literature, the guidelines regarding RBC transfusions for preterm neonates are controversial, with administration strategies varying greatly [20,21]. Due to such variations in administration protocols, the lack of a national guideline, and patients being admitted for up to one month, with rare cases in which the duration of hospitalization exceeds this period, rhEPO treatment was carried out according to the protocol of the “Louis Turcanu” Children’s Emergency Clinical Hospital. Iron was administered at a dose of 5 mg/kg BW/day, taking various factors into consideration, including breastfeeding versus formula, to prevent iron deficiency. Approval was granted on 5 October 2023 by the Ethics Committee for Scientific Research and Development of the “Louis Turcanu” Children’s Emergency Clinical Hospital Timișoara with approval protocol code number 84.

### 2.2. Reference Intervals and Categorized Variables

Reference intervals for LDH and serum EPO were 135–750 U/L [22] and 4–24 mIU/mL [23], respectively.

BW was categorized as “extremely low” (<1000 g), “very low” (<1500 g), and “low” (<2500 g), as per WHO guidelines [24].

GA categorization was also performed as per WHO guidelines [25]; namely, neonates with a GA under 28 weeks were classified as “extremely preterm”, those with a GA of 28 to less than 32 weeks were classified as “very preterm”, and those with a GA of 32 to 37 weeks were classified as “moderate to late preterm”. It is noteworthy that in our study, the 32-to-37-week group only contained neonates with a GA of up to 34 weeks; therefore, this group may present different pathophysiological characteristics compared to similar groups in other studies that include a proportionate number of neonates with GA of 35 to 37 weeks.

Apgar scores of 7–10 were considered “reassuring”, scores of 4–6 were considered “moderately abnormal”, while scores of 0–3 were considered “low”, as per the American College of Obstetrics and Gynecology [26].

PT and aPTT levels of 11–14 s and 23–35 s, respectively, were considered normal [27].

AOP severity was categorized based on WHO guidelines, using the 6–59 months interval in absence of a 0–6 months interval; therefore, cases with Hb levels of 100–109 g/L were classified as mild AOP, those with Hb levels of 70–99 g/L were classified as moderate AOP, and those with Hb levels less than <70 g/L were classified as severe AOP [28].

### 2.3. Statistical Analysis

All continuous variables have been represented as mean ± standard deviation (SD). Categorical variables have been represented as numbers or percentages. Univariately, Student’s *t*-test was used to examine continuous variables, while the one-sample binomial and χ2 tests were used to examine dichotomous variables. Independent nominal variables, such as sex, EPO administration, and iron administration, were dichotomized. Certain independent continuous variables such as 1 min Apgar score, aPTT, PT, LDH, and serum EPO were used only as scale variables, Hb, HCT, RBC were used both as scale variables and dichotomized variables, while GA, BW, and 5 min Apgar score were used both as scale variables and ranked categorical variables. Dependent variables such as transfusion administration, abnormal serum ferritin, and abnormal serum iron were dichotomized, while AOP was used both as a dichotomized variable and a ranked categorical variable (i.e., no AOP, mild AOP, moderate AOP, and severe AOP). Following univariate binomial logistic regression, factors with a statistically significant association to AOP or transfusion administration were further analyzed using multivariate binomial logistic regression. The odds ratios (ORs) and the adjusted ORs (AORs) have been calculated for univariate and multivariate analyses, respectively. Significant factors underwent multivariate multinominal logistic regression analysis to study the relationship between multiple independent factors and AOP severity. The relationship between rank-transformed (GA, BW, 5 min Apgar score) and dichotomized (sex, LDH, aPTT, PT, iron administration, EPO administration) data and the AOP stage was further studied using the Spearman ranked correlation and rank-biserial correlation coefficient, respectively. ANCOVA was used to further study the correlation between EPO administration and 21-day levels of Hb, HCT, and serum EPO. Statistical analyses were conducted using SPSS Version 23 (Chicago, IL, USA). All tests used in the statistical analyses were two-tailed. *p*-values < 0.05 were considered significant. 

## 3. Results

### 3.1. Distribution of Patients

In the current study, 108 neonates with GA ≤ 34 weeks and BW < 2500 g were included to study the effects that EPO administration in the first 7 days of life, iron administration during the 7th to 21st days of life, and other perinatal characteristics, including GA, BW, Apgar scores at 1 and 5 min, and LDH, Hb, HCT, RBC count, aPTT, PT, and serum EPO at 1 day of life have on various outcomes, including AOP (any severity), AOP severity, RBC count, Hb, HCT, and serum EPO, ferritin, and iron levels at 21 days of life and transfusion administration within the first 21 days of life.

The male to female ratio was 1.45 (64 males and 44 females), with no statistically significant difference being found between sexes in regard to GA (*p* = 0.33), BW (*p* = 0.79), 1- and 5 min Apgar scores (*p* = 0.098 and *p* = 0.171, respectively), RBC count (*p* = 0.078), aPTT (*p* = 0.96), PT (*p* = 0.18), LDH (*p* = 0.72), and serum EPO (*p* = 0.53) at 1 day of life. The only baseline characteristics which significantly differed were day-1 Hb (*p* < 0.01) and HCT (*p* < 0.05) levels. 

**Figure 1:**
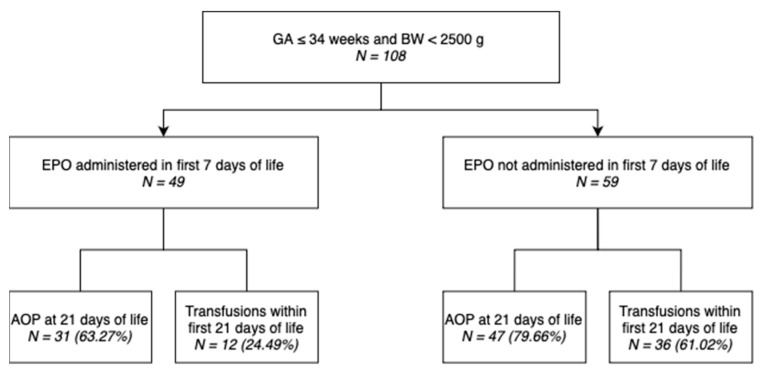
Distribution of neonates included in the study.

Out of 108 neonates included in this study, 49 (45.37%) underwent EPO administration within the first 7 days of life, as shown in the diagram (Figure 1).

Table 1 illustrates the AOP (any severity) incidence rates, as well as the incidence rates of its specific stages in different subgroups based on different categorized baseline variables of our study population, such as GA and BW, and provides descriptive information regarding the variation between them.

The newborns were distributed to different groups based on perinatal characteristics, such as sex, GA category, BW category, 1- and 5 min Apgar score categories, LDH within range, aPTT within range, PT within range, congenital anemia, and iron and EPO treatment. 

The majority of both males (78.13%) and females (63.64%) have been diagnosed with some AOP stage (mild, moderate, or severe). The incidence of AOP (any severity) gradually decreased as GA and BA increased: 88.89%, 76.36%, and 57.14% for extremely (<28 weeks), very (28–31 weeks), and moderate to late (32–37 weeks) preterm, and 85.71%, 74.47%, and 62.50% for extremely low (<1000 g), very low (1000–1499 g), and low (1500–2499 g) BW neonates, respectively. Similarly, there was a gradual decrease in the incidence of AOP (any severity) as 1 and 5 min Apgar scores increased: 81.82%, 76.47%, and 65.38% for 0–3, 4–6, and 7–10 1 min Apgar scores, and 100%, 75.56%, and 69.35% for 0–3, 4–6, and 7–10 5 min Apgar scores, respectively.

Neonates with pathological LDH, PT, and serum EPO levels on the first day of life showed higher AOP (any severity) incidence rates compared to those presenting levels within the reference ranges (85.87%, 78.52%, and 83.18%, respectively). 

Surprisingly, less neonates with pathological aPTT levels on the first day of life presented AOP (any severity) compared to those with normal aPTT levels (69.47% vs. 92.31%, respectively). 

Neonates that were administered iron during the 7th to 21st days of life showed a higher AOP (any severity) incidence rate compared to those that did not undergo iron treatment (80.00% vs. 62.50%), while neonates that underwent early EPO administration showed a lower AOP (any severity) incidence rate compared to those that did not undergo EPO treatment (63.27% vs. 79.66%).

Table 2 showcases the variation in incidence rates of RBC transfusions in different subgroups of the study population represented by categorized baseline variables, such as GA and BW.

Half of the male neonates required transfusions within the first three weeks of life, compared to only 36.36% of female neonates.

The need for transfusions within the first three weeks of life also gradually decreased as GA and BA increased: 77.78%, 47.27%, and 22.86% for extremely preterm, very preterm, and moderate to late preterm, and 76.19%, 51.06%, and 20.00% for extremely low, very low, and low BW neonates, respectively.

Neonates with pathological PT levels at 1 day of life required transfusions within the first three weeks of life compared to those with normal PT levels (46.97% vs. 40.48%, respectively). 

Surprisingly, less neonates with pathological aPTT levels on the first of life required transfusions within the first 21 days of life (42.11% vs. 61.54%, respectively). 

Neonates that were administered iron during the 7th to 21st days of life showed a higher transfusion incidence rate compared to those that did not undergo iron treatment (60.00% vs. 25.00%, respectively), while neonates that were administered EPO during the first week of life showed a lower transfusion incidence rate compared to those that did not undergo EPO treatment (24.49% vs. 61.02%, respectively).

### 3.2. Univariate Perinatal Characteristic Comparison

Perinatal characteristics of neonates who underwent and did not undergo EPO administration have been listed in Table 3. This analysis served to identify any considerable differences between the intervention and control groups that might have an impact on the subsequent analysis and results. The groups administered EPO within 7 days and not administered EPO within 7 days did not differ at baseline in terms of sex, GA, BW, Apgar scores at 1 and 5 min, Hb, HCT, RBC count, aPTT, PT, LDH, or AOP at 1 day of life. The serum EPO levels at 1 days of life was the only perinatal characteristic that was significantly different between neonates that received EPO treatment within the first 7 days of life and those that were not administered EPO within the first 7 days of life.

### 3.3. Univariate Risk Factor Comparison

#### 3.3.1. AOP (Any Severity)

As indicated in Table 4, the group with AOP at 21 days of life and the group without AOP at 21 days of life did not differ at baseline in terms of sex, Apgar scores at 1 and 5 min, aPTT, PT, LDH, or serum EPO at 1 day of life, but did significantly differ in terms of GA, BW, Hb, HCT, RBC count, and iron administration. The difference between the groups of neonates administered and not administered EPO was nearly significant, at *p* = 0.058, prompting further investigation using logistic regression. The difference between the groups of neonates administered and not administered iron was statistically significant, with the former being more likely to present AOP at 21 days of life (*p* < 0.001).

#### 3.3.2. Transfusions

As presented in Table 5, the group with transfusions within first three weeks of life and the group without transfusions within first three weeks of life differed at baseline in terms of GA, BW, Apgar scores at 1 min, Hb, HCT, RBC count, serum EPO at 1 day of life, and EPO administration.

Table 4 and Table 5 display the results of Student’s *t*-tests and Chi-square tests for continuous or binary variables, respectively, which was the first level of analysis for determining whether there is an association between rhEPO administration, as well as other baseline factors and AOP and transfusion incidence.

### 3.4. Univariate Analysis of Risk Factors

#### 3.4.1. AOP (Any Severity)

As indicated in Table 6, GA, BW, Hb, HCT, and RBC count have been found to be significant protective factors against AOP at 21 days, while iron administration has been identified as a significant AOP risk factor.

#### 3.4.2. Transfusions

As presented in Table 7, GA, BW, HCT level, RBC count and EPO administration have been found to be significant protective factors against transfusion within three weeks of life, while serum EPO has identified as a statistically significant risk factors for transfusions within 21 days (*p* < 0.001).

Table 6 and Table 7 represent the results of univariate logistical regression, showing the ORs of each baseline factors and of rhEPO treatment in regard to developing AOP. It was important to analyze all the available baseline factors as these were all potential covariates in the next level of statistical analysis, namely multivariate logistical regression, which is used to calculate the adjusted ORs based on different baseline factors that could have impacted the results.

### 3.5. Multivariate Analysis of Risk Factors

#### 3.5.1. Multivariate Associations with AOP (Any Severity)

Factors that demonstrated a statistically significant correlation to AOP in the previous analysis, namely GA, BW, Hb at 1 day of life, HCT at 1 day of life, RBC count at 1 day of life, and iron administration, were further studied using multivariate binomial logistic regression analysis, and their AORs were calculated. The analysis was performed following the same variable classification used in the above tests.

As indicated in Table 8, unlike in univariate logistic regression analysis, the only factor that remains statistically significant is iron administration between the 7th and 21st day of life, with *p* < 0.05. Therefore, iron administration is shown to increase the risk of neonates developing AOP at 21 days of age 2.7-fold, having an AOR of 2.75 (95% CI, 1.06–7.11). However, this wide confidence interval warrants further research involving larger data sets.

#### 3.5.2. Multinomial Associations with Specific AOP Stages

Factors that were significantly correlated to AOP above were then analyzed using multinomial (ordinal/ranked dependent variable) logistic regression. The analysis was performed following the variable classification used in above, but instead of using the dichotomous any-severity AOP variable, the AOP category variable (ranked) was used to determine which correlation between multiple factors and AOP stages/levels was the highest. Table 9 showcases the results of the multinomial logistical regression, which were used to analyze the relationship between different factors and the incidence of different AOP stages.

As presented in Table 9, the first set of coefficients represents comparisons between neonates with Absence of AOP at 21 days of life (coded 0) and those with Mild AOP at 21 days of life (coded 1 in this portion of the output). None of the above factors were significant predictors in the model, as all *p*-values > 0.05. 

The second set of coefficients represents comparisons between neonates with Absence of AOP at 21 days of life (coded 0) and those with Moderate AOP at 21 days of life (coded 2 in this portion of the output). Only iron administration in the 7th to 21st day of life was a significant predictor (b = 1.27, s.e. = 0.52, *p* < 0.05) in the model, as neonates that underwent iron administration were more 3.5-fold more likely to have moderate AOP that those who did not undergo iron administration, with an OR of 3.56 (95% CI, 1.28–9.93).

The final set of coefficients represents comparisons between neonates with Absence of AOP at 21 days of life (coded 0) and those with Severe AOP at 21 days of life (coded 3 in this portion of the output). As there was only 1 case of Severe AOP, there was insufficient data to test this set.

**Figure 2:**
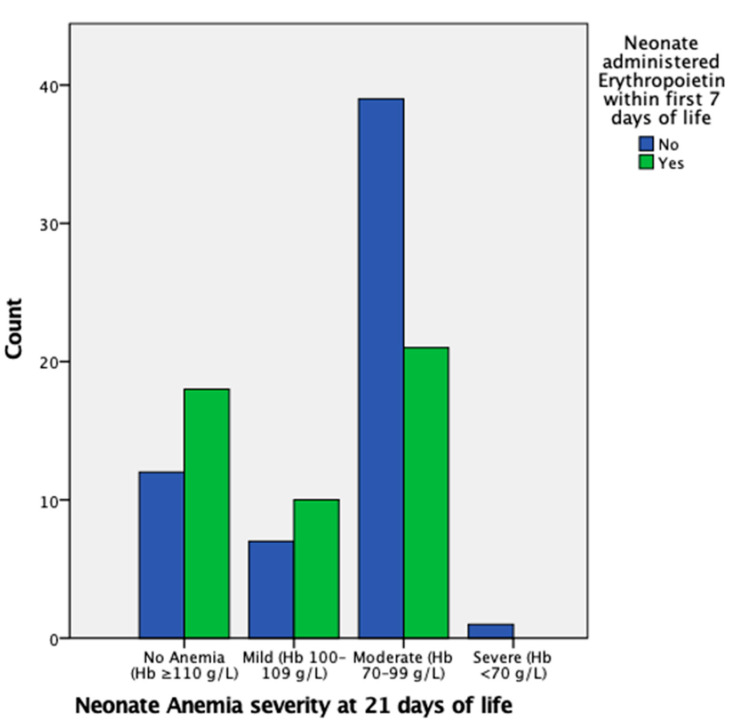
Proportion of neonates with mild, moderate, severe, or no AOP at 21 days of life grouped by administration of EPO in the first week of life. The proportion of neonates that had moderate AOP at 21 days of life is markedly higher in the group that did not undergo EPO administration.

EPO administration in the first week of life is insignificant in regard to mild AOP at 21 days of life; however, it is significant in reducing the likelihood of moderate AOP at 21 days of life, with an OR of 0.36 (95% CI, 0.15–0.89) and *p* < 0.05, as also indicated in Figure 2. There were insufficient data for severe AOP at 21 days of life.

#### 3.5.3. Spearman Ranked Correlations to Specific AOP Stages

The relationship between particular AOP stages and either rank-transformed GA (32–37, 28–31, and <28 weeks), BW (1500–2499, 1000–1499, and <1000 g), Apgar score at 5 min (7–10, 4–6, and 0–3), and dichotomized data (Sex, Normal LDH, Normal aPTT, Normal PT, Iron administration, and EPO administration) was further studied using either the Spearman ranked or rank biserial correlation coefficients, respectively.

In regard to GA category, there was a strong (ρ = 0.67), statistically significant (*p* < 0.001) correlation between GA category and BW category (*p* < 0.001), a “near strong” (ρ = 0.42), statistically significant (*p* < 0.001) correlation between GA category and 5 min Apgar score category, and a moderate (ρ = −0.32), statistically significant (*p* = 0.001) negative correlation (−0.314) between GA category and AOP severity at 21 days of life. 

In regard to BW category, there was a strong (ρ = 0.67), statistically significant (*p* < 0.001) correlation between BW category and GA category, a moderate (ρ = 0.36), statistically significant (*p* < 0.001) correlation between BW category and Apgar score (5 min) category, and a moderate (ρ = −0.28), significant (*p* < 0.01) negative correlation between BW category and AOP severity at 21 days of life. 

In regard to the 5 min Apgar score category, there was a “near strong” (ρ = 0.42), statistically significant (*p* < 0.001) correlation between 5 min Apgar score category and GA category, a moderate (ρ = 0.36), statistically significant (*p* < 0.001) correlation between 5 min Apgar score category and BW category, and a weak negative correlation (ρ = −0.10), statistically unsignificant (*p* > 0.05) negative correlation between 5 min Apgar score category and AOP severity at 21 days of life.

Therefore, following the Spearman ranked analysis, it was demonstrated that GA had a stronger negative correlation with AOP stage/level than BW (ρ = −0.31 vs. ρ = −0.28), but that it was only a moderate correlation.

#### 3.5.4. Multivariate Associations with Transfusion Administration

Significant factors that were correlated to transfusions in the previous univariate regression analysis, i.e., GA, BW, Hb at 1 day of life, HCT at 1 day of life, RBC count at 1 day of life, serum EPO at 1 day of life, and EPO administration, were studied using multivariate regression and their AORs were calculated. The analysis was performed following the same variable classification used in the above tests.

As indicated in Table 10, unlike in univariate logistic regression analysis, no factor remained significant (*p* < 0.05), with BW being the only “near significant” factor, with a *p*-value of exactly 0.05 and an AOR of 0.998 (95% CI, 0.996–1.000).

### 3.6. ANCOVA of Hb, HCT, and Serum EPO Levels at 21 Days of Life following EPO Administration

To further study the EPO administration in regard to its impact on AOP incidence, the relationships between EPO treatment in the first week of life and the Hb, HCT, and serum EPO levels of neonates at 21 days of life were tested using ANCOVA, with Hb, HCT, and serum EPO levels at 21 days of life as dependent continuous variables, EPO administration as an independent variable, and GA, BW, and Apgar scores at 1 day, as well as Hb, HCT, RBC, LDH, and serum EPO levels, and aPTT and PT as covariate candidates. Table 11 displays the ANCOVA test results, which were used to analyze the differences in Hb, HCT and serum EPO at 21 days between the intervention and control group.

As indicated in Table 11, although EPO administration in the first 7 days of life was shown to only have a statistically significant relationship with moderate AOP at 21 days of life and not with any-stage AOP at 21 days of life in the above regression analyses, the above ANCOVA results show that EPO administration in the first week of life is significantly correlated with elevated Hb, HCT, and serum EPO levels at 21 days of life.

## 4. Discussion

The results obtained in this study point out well-known previously researched benefits of rhEPO administration, as well as considerable differences.

RBC transfusions, which have been previously correlated to both an increased morbidity and an increased mortality [6], were required in 48 out of 108 neonates included in this study; however, the proportion of neonates that did not undergo rhEPO therapy and required RBC transfusions was more than double that of neonates that underwent rhEPO treatment and required transfusions.

Similar to the results of a recent Cochrane review [15], a reduction in the number of transfusions required has been noted in this study following univariate logistic analysis (OR: 0.21, 95% CI, 0.09–0.48). This, however, contradicts the findings of Doyle J.J. (1997) [9], in which, dissimilar to rhEPO administration starting from the 21st day of life, rhEPO administration starting at less than one week of life was not associated with a decreased number of transfusions. Interestingly, following a multivariate analysis performed in this study, rhEPO therapy was no longer associated with a decrease in transfusions (AOR: 0.24, 95% CI, 0.04–1.33).

Alongside a reduction in the number of transfusions required, increases in Hb and HCT have also been reported in the literature [29]. In this study, ANCOVA revealed that rhEPO administration in the first week of life is significantly correlated with elevated levels of both Hb (η^2^ = 0.1, *p* < 0.01) and HCT (η^2^ = 0.07, *p* < 0.01) at 21 days of life. Furthermore, the correlation found between rhEPO treatment and 21-day serum levels (η^2^ = 0.20) confirms rhEPO is an erythropoietic growth factor, explaining 20% of the variation in 21-day serum levels in this study.

Although univariate regression analysis showed only a near-significant association between rhEPO administration and AOP (*p* = 0.061), multivariate logistic regression analysis revealed a significant relationship between rhEPO therapy and moderate AOP (OR: 0.36, 95% CI, 0.15–0.89), the most common AOP stage found in this study. This near three-fold reduction in moderate AOP incidence is consistent with a large majority of studies that recommend rhEPO administration and point out multiple benefits of this treatment [1,9,10,11,12,13,14,15,16,18,19]. Furthermore, this warrants future investigation into the effects rhEPO has on different levels of AOP.

Surprisingly, iron administration within the first 7–21 days of life was the only statistically significant AOP risk factor when undergoing multivariate logistical analysis (AOR: 2.75, 95% CI, 1.06–7.11), albeit a wide CI that warrant further studies involving larger sample sizes. This near three-fold increase has not been previously reported in the literature and may be statistically insignificant in larger study populations.

An increase in Hb was seen primarily after three weeks of rhEPO treatment, and no significant differences in the RBC transfusion volume between neonates were detected.

Unfortunately, newborns with extremely low BW, who present more serious health issues and have an increased need for RBC transfusions postnatally, do not consistently respond to rhEPO therapy. In addition, younger GA and age at time of anesthesia, as well as the need for surgery in preterm newborns, are also significantly correlated with both increased morbidity and mortality, further complicating the clinical decision-making required in such cases with multiple co-morbidities [8]. The complexity and high number of factors related to treating premature and underweight neonates, alongside the variations in transfusion practices and triggering thresholds [6,7], call forth the need to optimize current clinical guidelines and practices.

This study includes multiple limitations. The main limitations of the study are the lack of randomization, which could not be performed due to ethical considerations, and, as a result of administering rhEPO only to neonates with EPO levels under 4.3 mUI/mL on the first day of life, the lower serum EPO levels of neonates in the intervention group compared to the control group. Another major limitation of the study is represented by the small number of patients included, which warrants further studies on the effects of rhEPO therapy. Another significant limitation is the lack of a national guideline covering rhEPO administration, leading to a non-standard administration protocol adopted by the “Louis Turcanu” Children’s Emergency Clinical Hospital Timisoara in order to compensate for the fact that patients are only admitted for up to one month, with the vast majority of them being lost to follow-up. Loss to follow-up is a major limitation in itself, which may be higher than in other studies due to the relatively large rural environment, low socioeconomic status, and low level of health education in the region served by the hospital. Moreover, the lack of data regarding various morbidities of the preterm infants, including RDS, infection, IVH, is a considerable limitation. Another limitation is the lack of hypothermia data.

As this is one of the few studies analyzing AOP in an East-European population of preterm and low birthweight neonates, specifically West Romania, the results can be included in future meta-analyses alongside other studies investigating the relationship between rhEPO treatment and AOP to account for variation of effect sizes across different populations.

## 5. Conclusions

In this study, rhEPO treatment within the first 7 days of life was confirmed to reduce moderate AOP incidence and increase Hb, HCT, and serum EPO levels at 21 days of life. Additionally, rhEPO therapy has been significantly associated with reduced incidence of transfusions within the first 21 days of life. These results underline the multiple benefits of rhEPO treatment in preterm neonates.

## Figures and Tables

**Table 1:** Distributions of AOP (any severity), mild AOP, moderate AOP, and severe AOP groups by perinatal characteristics.

				AOP Severity at 21 Days of Life					
		AOP at 21 Days of Life		Mild		Moderate		Severe	
Subgrouping	Total Population (*n*)	No. of Neonates	Percentage	No. of Neonates	Percentage	No. of Patients	Percentage	No. of Patients	Percentage
Total	108	78	72.22%	17	15.74%	60	55.56%	1	0.93%
Sex									
Male	64	50	78.13%	7	10.94%	43	67.19%	0	0.00%
Female	44	28	63.64%	10	22.73%	17	38.64%	1	2.27%
Gestational age (wks)									
32–37	35	20	57.14%	7	20.00%	13	37.14%	0	0.00%
28–31	55	42	76.36%	9	16.36%	32	58.18%	1	1.82%
<28	18	16	88.89%	1	5.56%	15	83.33%	0	0.00%
Body Weight (g)									
1500–2499	40	25	62.50%	9	22.50%	16	40.00%	0	0.00%
1000–1499	47	35	74.47%	6	12.77%	29	61.70%	0	0.00%
<1000	21	18	85.71%	2	9.52%	15	71.43%	1	4.76%
Apgar 1 min									
7–10	52	34	65.38%	6	11.54%	28	53.85%	0	0.00%
4–6	34	26	76.47%	8	23.53%	17	50.00%	1	2.94%
0–3	22	18	81.82%	3	13.64%	15	68.18%	0	0.00%
Apgar 5 min									
7–10	62	43	69.35%	10	16.13%	33	53.23%	0	0.00%
4–6	45	34	75.56%	7	15.56%	26	57.78%	1	2.22%
0–3	1	1	100.00%	0	0.00%	1	100.00%	0	0.00%
Normal aPTT at 1 day of life (23–35 s)									
Yes	13	12	92.31%	0	0.00%	12	92.31%	0	0.00%
No	95	66	69.47%	17	17.89%	48	50.53%	1	1.05%
Normal Prothrombin Time 1 day of life (11–14 s)									
Yes	42	28	66.67%	7	16.67%	21	50.00%	0	0.00%
No	66	50	75.76%	10	15.15%	39	59.09%	1	1.52%
Normal LDH at 1 day of life (135–750 U/L)									
Yes	60	42	70.00%	12	20.00%	30	50.00%	0	0.00%
No	48	36	75.00%	5	10.42%	30	62.50%	1	2.08%
Iron administered within 7–21 days of life									
Yes	60	4	57.14%	1	14.29%	3	42.86%	0	0.00%
No	48	74	73.27%	16	15.84%	57	56.44%	1	0.99%
Congenital anemia									
Yes	7	31	63.27%	10	20.41%	21	42.86%	0	0.00%
No	101	47	79.66%	7	11.86%	39	66.10%	1	1.69%

Abbreviations: AOP, anemia of prematurity; aPTT, activated partial thromboplastin time; LDH, lactate dehydrogenase.

**Table 2:** Distributions of transfusion groups by perinatal characteristics.

		Transfusions within 21 Days of Life	
Subgrouping	Total Population (*n*)	No. of Neonates	Percentage
Total	108	48	44.44%
Sex			
Male	64	32	50.00%
Female	44	16	36.36%
Gestational age (wks)			
32–37	35	8	22.86%
28–31	55	26	47.27%
<28	18	14	77.78%
Body Weight (g)			
1500–2499	40	8	20.00%
1000–1499	47	24	51.06%
<1000	21	16	76.19%
Apgar 1 min			
7–10	52	19	36.54%
4–6	34	15	44.12%
0–3	22	14	63.64%
Apgar 5 min			
7–10	62	24	38.71%
4–6	45	23	51.11%
0–3	1	1	100.00%
Normal aPTT at 1 day of life (23–35 s)			
Yes	13	8	61.54%
No	95	40	42.11%
Normal Prothrombin Time 1 day of life (11–14 s)			
Yes	42	17	40.48%
No	66	31	46.97%
Iron administered within 7–21 days of life			
Yes	60	36	60.00%
No	48	12	25.00%
Congenital anemia			
Yes	7	5	71.43%
No	101	43	42.57%
EPO administered in first 7 days			
Yes	49	12	24.49%
No	59	36	61.02%
Normal LDH at 1 day of life (135–750 U/L)			
Yes	60	28	46.67%
No	48	20	41.67%

Abbreviations: aPTT—activated partial thromboplastin time; EPO—erythropoietin; LDH—lactate dehydrogenase.

**Table 3:** Differences in perinatal characteristics between neonates who underwent EPO administration in the first 7 days of life and those who did not.

Characteristics	EPO Administered within 7 Days of Life (*n* = 49)	EPO not Administered within 7 Days of Life (*n* = 59)	*p* Value
Male	26 (53.06%)	38 (64.41%)	0.169
Female	23 (46.94%)	21 (35.59%)	0.880
GA, weeks	29.71 ± 2.88	30.12 ± 2.31	0.420
BW, g	1365.41 ± 470.54	1405.15 ± 397.38	0.635
Apgar 1 min	5.80 ± 2.04	5.53 ± 2.51	0.538
Apgar 5 min	6.53 ± 1.66	6.58 ± 1.38	0.876
Hb at 1 day of life, g/dL	15.52 ± 3.23	15.22 ± 2.51	0.585
HCT at 1 day of life, %	46.01 ± 6.96	43.72 ± 6.77	0.086
RBC count at 1 day of life, million/μL	4.28 ± 0.63	4.13 ± 0.63	0.214
aPTT at 1 day of life, s	56.06 ± 37.75	52.83 ± 19.60	0.569
PT at 1 day of life, s	15.78 ± 5.43	16.36 ± 4.42	0.541
LDH at 1 day of life, U/L	726.67 ± 368.84	804.62 ± 396.23	0.296
Serum EPO at 1 day of life, mIU/mL	2.25 ± 1.06	9.41 ± 5.00	<0.001
AOP at 1 day of life	4 (8.16%)	3 (5.08%)	1.000

Abbreviations: EPO—erythropoietin; GA—gestational age; BW—body weight; Hb—hemoglobin; HCT—hematocrit; RBC—red blood cell; aPTT—activated partial thromboplastin time; PT—prothrombin time; LDH—lactate dehydrogenase; AOP—anemia of prematurity. Categorical variables are represented as *n* (%); continuous variables are displayed as means ± SD.

**Table 4:** Univariate analysis of baseline factors in neonates that developed AOP (any severity) at 21 days of life and those who did not.

Characteristics	Without AOP at 21 Days of Life (*n* = 30)	With AOP at 21 Days of Life (*n* = 78)	*p* Value
Male	14 (46.67%)	50 (64.10%)	<0.001
Female	16 (53.33%)	28 (35.90%)	0.097
GA, weeks	30.90 ± 2.26	29.56 ± 2.61	<0.05
BW, g	1526.83 ± 434.50	1333.38 ± 419.38	<0.05
Apgar 1 min	6.27 ± 1.95	5.41 ± 2.39	0.083
Apgar 5 min	6.97 ± 1.56	6.40 ± 1.46	0.078
Hb at 1 day of life, g/dL	16.46 ± 3.01	14.94 ± 2.68	<0.05
HCT at 1 day of life, %	47.00 ± 8.56	43.90 ± 6.02	<0.05
RBC count at 1 day of life, million/μL	4.48 ± 0.76	4.09 ± 0.54	<0.01
aPTT at 1 day of life, s	58.76 ± 47.02	52.58 ± 18.39	0.326
PT at 1 day of life, s	15.01 ± 4.45	16.52 ± 5.01	0.151
LDH at 1 day of life, U/L	808.17 ± 445.65	754.29 ± 359.95	0.517
Serum EPO at 1 day of life, mIU/mL	4.77 ± 4.05	6.70 ± 5.49	0.083
Iron administered during 7th–21st day of life	12 (40.00%)	48 (61.54%)	<0.001
EPO administered in first 7 days of life	3 (10.00%)	4 (5.13%)	0.058

Abbreviations: AOP—anemia of prematurity; GA—gestational age; BW—body weight; Hb—hemoglobin; HCT—hematocrit; RBC—red blood cell; aPTT—activated partial thromboplastin time; PT—prothrombin time; LDH—lactate dehydrogenase; EPO—erythropoietin. Categorical variables are represented as *n* (%); continuous variables are displayed as means ± SD.

**Table 5:** Univariate analysis of baseline factors in neonates that required transfusions within the first three weeks of life and those who did not.

Characteristics	Transfusions Not Required in First 21 Days of Life (*n* = 60)	Transfusions Required in First 21 Days of Life (*n* = 48)	*p* Value
Male	32 (53.30%)	32 (66.67%)	1.000
Female	28 (46.67%)	16 (33.30%)	0.970
GA, weeks	30.87 ± 2.31	28.77 ± 2.44	<0.001
BW, g	1539.75 ± 423.64	1196.33 ± 360.12	<0.001
Apgar 1 min	6.08 ± 2.00	5.10 ± 2.55	<0.05
Apgar 5 min	6.80 ± 1.49	6.25 ± 1.48	0.059
Hb at 1 day of life, g/dL	16.16 ± 2.88	14.36 ± 2.49	<0.01
HCT at 1 day of life, %	47.15 ± 5.96	41.76 ± 6.92	<0.001
RBC count at 1 day of life, million/μL	4.42 ± 0.54	3.93 ± 0.64	<0.001
aPTT at 1 day of life, s	53.72 ± 34.14	55.02 ± 21.71	0.819
PT at 1 day of life, s	15.77 ± 4.75	16.51 ± 5.07	0.432
LDH at 1 day of life, U/L	783.17 ± 371.03	751.88 ± 403.45	0.676
Serum EPO at 1 day of life, mIU/mL	4.28 ± 2.91	8.51 ± 6.35	<0.001
EPO administered in first 7 days of life	37 (61.67%)	12 (25.00%)	<0.01

Abbreviations: GA—gestational age; BW—body weight; Hb—hemoglobin; HCT—hematocrit; RBC—red blood cell; aPTT—activated partial thromboplastin time; PT—prothrombin time; LDH—lactate dehydrogenase; EPO—erythropoietin. Categorical variables are represented as *n* (%); continuous variables are displayed as means ± SD.

**Table 6:** Results of univariate logistic regression incorporating factors potentially associated with AOP.

Characteristics	OR (95% CI)	*p* Value	AOP Incidence Rate *
Sex, male/female	0.490 (0.209–1.150)	0.101	78.13%/63.64%
GA	0.802 (0.668–0.963)	<0.05	
BW	0.999 (0.999–1.000)	<0.05	
Apgar score at 1 min	0.836 (0.680–1.026)	0.087	
Apgar score at 5 min	0.765 (0.566–1.034)	0.081	
Hb at 1 day of life	0.776 (0.635–0.949)	<0.05	
HCT at 1 day of life	0.930 (0.868–0.997)	<0.05	
RBC count at 1 day of life	0.316 (0.139–0.716)	<0.01	
aPTT at 1 day of life	0.993 (0.980–1.007)	0.356	
PT at 1 day of life	1.083 (0.970–1.208)	0.155	
LDH at 1 day of life	1.000 (0.999–1.001)	0.514	
Serum EPO at 1 day of life	1.093 (0.986–1.212)	0.090	
Iron administered during 7th–21st day of life, yes/no	2.400 (1.014–5.678)	<0.05	80.00%/62.50%
EPO administered in first 7 days of life, yes/no	0.440 (0.186–1.039)	0.061	63.27%/79.66%

Abbreviations: OR—odds ratio; AOP—anemia of prematurity; GA—gestational age; BW—body weight; Hb—hemoglobin; HCT—hematocrit; RBC—red blood cell; aPTT—activated partial thromboplastin time; PT—prothrombin time; LDH—lactate dehydrogenase; EPO—erythropoietin. * AOP incidence rate if neonate depending on sex and whether they underwent iron administration during the 7th to 21st day of life and EPO administration within the first 7 days of life.

**Table 7:** Results of univariate logistic regression incorporating factors potentially associated with transfusion administration.

Characteristics	OR (95% CI)	*p* Value	Transfusion Incidence Rate *
Sex, male/female	1.750 (0.798–3.840)	0.163	50.00%/36.36%
GA	0.694 (0.578–0.833)	<0.001	
BW	0.998 (0.997–0.999)	<0.001	
Apgar score at 1 min	0.827 (0.696–0.982)	<0.05	
Apgar score at 5 min	0.779 (0.599–1.012)	0.062	
Hb at 1 day of life	0.762 (0.640–0.907)	<0.01	
HCT at 1 day of life	0.873 (0.812–0.938)	<0.001	
RBC count at 1 day of life	0.230 (0.105–0.502)	<0.001	
aPTT at 1 day of life	1.002 (0.989–1.015)	0.818	
PT at 1 day of life	1.032 (0.954–1.117)	0.432	
LDH at 1 day of life	1.000 (0.999–1.001)	0.673	
Serum EPO at 1 day of life	1.230 (1.103–1.372)	<0.001	
EPO administered in first 7 days of life, yes/no	0.207 (0.090–0.478)	<0.001	24.49%/61.02%

Abbreviations: OR—odds ratio; GA—gestational age; BW—body weight; Hb—hemoglobin; HCT—hematocrit; RBC—red blood cell; aPTT—activated partial thromboplastin time; PT—prothrombin time; LDH—lactate dehydrogenase; EPO—erythropoietin. * AOP incidence rate of neonate depending on sex and whether they underwent EPO administration within the first 7 days of life.

**Table 8:** Results of multivariate logistic regression analysis incorporating factors potentially associated with AOP (any severity).

Characteristics	AOR (95% CI)	*p* Value
GA	0.952 (0.706–1.283)	0.745
BW	0.999 (0.998–1.001)	0.401
Hb at 1 day of life	0.669 (0.295–1.515)	0.335
HCT at 1 day of life	1.170 (0.864–1.585)	0.310
RBC count at 1 day of life	0.351 (0.073–1.698)	0.193
Iron administration during 7th–21st day of life	2.749 (1.064–7.106)	<0.05

Abbreviations: AOR—adjusted odds ratio; GA—gestational age; BW—body weight; Hb—hemoglobin; HCT—hematocrit; RBC—red blood cell.

**Table 9:** Neonates without AOP compared to those with mild, moderate, and severe AOP in regard to various baseline factors.

Characteristics	OR (95% CI)	*p* Value
**Mild AOP**		
GA	1.087 (0.719–1.644)	0.691
BW	1.000 (0.998–1.002)	0.908
Hb at 1 day of life	0.640 (0.270–1.519)	0.312
HCT at 1 day of life	1.194 (0.854–1.671)	0.300
RBC count at 1 day of life	0.271 (0.031–2.345)	0.236
Iron administered during 7th–21st day of life	1.348 (0.373–4.880)	0.649
**Moderate AOP**		
GA	0.912 (0.667–1.247)	0.564
BW	0.999 (0.997–1.001)	0.285
Hb at 1 day of life	0.704 (0.307–1.614)	0.407
HCT at 1 day of life	1.154 (0.847–1.571)	0.364
RBC count at 1 day of life	0.366 (0.071–1.879)	0.228
Iron administered during 7th–21st day of life	3.564 (1.279–9.927)	0.015
Severe AOP *		

Abbreviations: OR—adjusted odds ratio; GA—gestational age; BW—body weight; Hb—hemoglobin; HCT—hematocrit; RBC—red blood cell. * Analysis could not be performed due to lack of data, i.e., only one neonate presented severe AOP at 21 days of life.

**Table 10:** Results of multivariate logistic regression analysis incorporating factors potentially associated with transfusion administration.

Characteristics	AOR (95% CI)	*p* Value
GA	0.864 (0.619–1.204)	0.387
BW	0.998 (0.996–1.000)	0.050
Apgar score at 1 min	0.913 (0.714–1.167)	0.467
Hb at 1 day of life	0.850 (0.581–1.243)	0.401
HCT at 1 day of life	0.944 (0.785–1.136)	0.544
RBC count at 1 day of life	0.695 (0.134–3.611)	0.665
Serum EPO at 1 day of life	1161 (0.964–1.398)	0.117
EPO administered in first 7 days of life, yes/no	0.236 (0.042–1.333)	0.102

Abbreviations: AOR—adjusted odds ratio; GA—gestational age; BW—body weight; Hb—hemoglobin; HCT—hematocrit; RBC—red blood cell.

**Table 11:** ANCOVA results showing percentage of variance in Hb, HCT, and serum EPO levels at 21 days of life explained by EPO administration in the first 7 days of life and the respective statistical significance.

		Hb Level at 21 Days of Life	HCT Level at 21 Days of Life	Serum EPO at 21 Days of Life
EPO administration in first 7 days of life	Percentage of variance explained	10 **	6.7 **	20.1 **
	*p*-value	<0.01	<0.05	<0.001

** Result is statistically significant, *p* < 0.05.

## Data Availability

All the data presented throughout this study are available on request and have not been publicized following European Union General Data Protection Regulation requirements for limiting the availability of sensitive personal information.
